# New Labdane-Type Diterpenoids and Anti-Inflammatory Constituents from *Hedychium coronarium*

**DOI:** 10.3390/ijms140713063

**Published:** 2013-06-25

**Authors:** Jih-Jung Chen, Chia-Wei Ting, Yi-Chin Wu, Tsong-Long Hwang, Ming-Jen Cheng, Ping-Jyun Sung, Tai-Chi Wang, Jinn-Fen Chen

**Affiliations:** 1 Graduate Institute of Pharmaceutical Technology & Department of Pharmacy, Tajen University, Pingtung 907, Taiwan; E-Mails: pye_wu@hotmail.com (Y.-C.W.); tcwang@mail.tajen.edu.tw (T.-C.W.); 2 Faculty of Pharmacy, College of Pharmacy, Kaohsiung Medical University, Kaohsiung 807, Taiwan; E-Mail: nestle0345@hotmail.com; 3 Graduate Institute of Natural Products, Chang Gung University, Taoyuan 333, Taiwan; E-Mail: htl@mail.cgu.edu.tw; 4 Bioresource Collection and Research Center (BCRC), Food Industry Research and Development Institute (FIRDI), Hsinchu 300, Taiwan; E-Mail: cmj0404@gmail.com; 5 National Museum of Marine Biology and Aquarium, Pingtung 944, Taiwan; E-Mail: pjsung@nmmba.gov.tw; 6 Taitung District Agricultural Research and Extension Station, Taitung 950, Taiwan; E-Mail: jfchen@mail.ttdares.gov.tw

**Keywords:** *Hedychium coronarium*, Zingiberaceae, labdane-type diterpenoid, anti-inflammatory activity

## Abstract

Four new labdane-type diterpenoids: hedychicoronarin (**1**), peroxycoronarin D (**2**), 7β-hydroxycalcaratarin A (**3**), and (*E*)-7β-hydroxy-6-oxo-labda-8(17),12-diene-15,16-dial (**4**), have been isolated from the rhizomes of *Hedychium coronarium*, together with 13 known compounds (**5**–**17**). The structures of these new compounds were determined through spectroscopic and MS analyses. Compounds **3**, **5**, **6**, and **10** exhibited inhibition (IC_50_ values ≤4.52 μg/mL) of superoxide anion generation by human neutrophils in response to formyl-L-methionyl-L-leucyl-L-phenylalanine/cytochalasin B (fMLP/CB). Compounds **3**–**6**, **10**, and **11** inhibited fMLP/CB-induced elastase release with IC_50_ values ≤6.17 μg/mL.

## 1. Introduction

*Hedychium coronarium* Koenig (Zingiberaceae) is a perennial herb distributed in India, Southeast Asian countries, southern China, Japan, and Taiwan [1]. *H. coronarium*, popularly called “White Butterfly Flower” or “Butterfly Ginger”, is used as a folk medicine for treatment of headache, contusion, inflammation, insomnia, stomach disorders, and sharp pain due to rheumatism in China [2,3].

Labdane-type diterpenes [3–9], farnesane-type sesquiterpenes [2], and their derivatives were isolated from this plant in previous studies. Many of these compounds were found to exhibit antiallergic [2], cytotoxic [3,4,9], anti-inflammatory [6,7], and hepatoprotective [8] activities. Granule proteases (e.g., elastase, cathepsin G, and proteinase-3) and reactive oxygen species (ROS) (e.g., superoxide anion (O_2_^•−^) and hydrogen peroxide) produced by human neutrophils are involved in the pathogenesis of a variety of inflammatory diseases.

**Figure 1:**
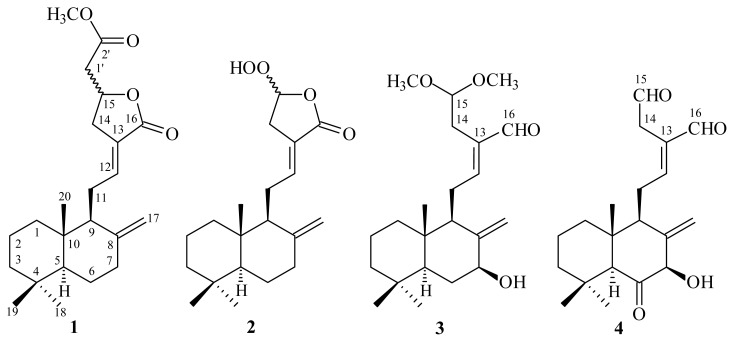
The chemical structures of new compounds **1**–**4** isolated from *H. coronarium*.

**Figure 2:**
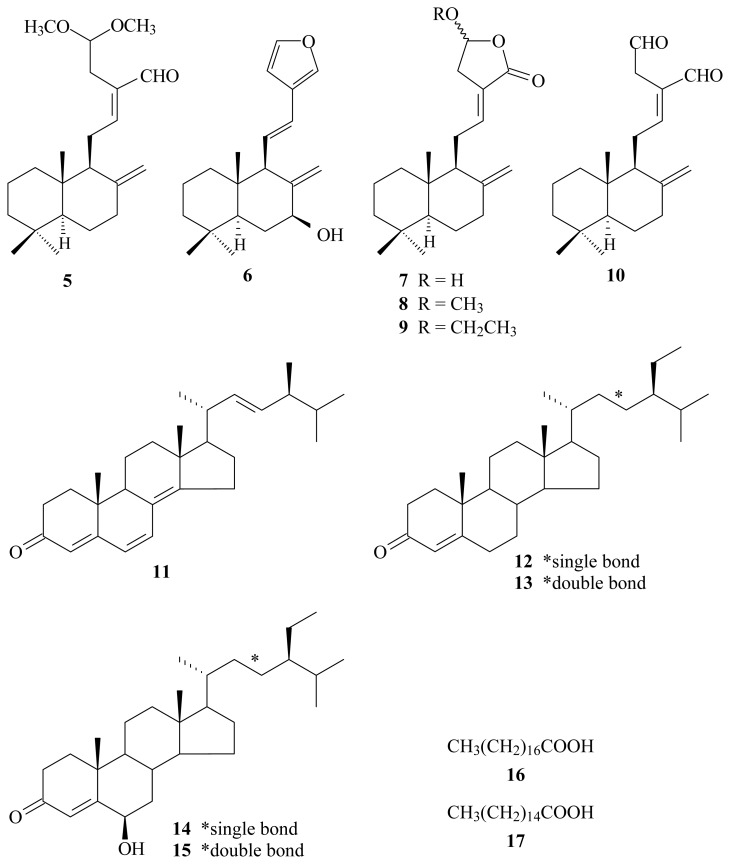
The chemical structures of known compounds **5**–**17** isolated from *H. coronarium*.

In our studies on the anti-inflammatory constituents of Formosan plants, many species have been screened for *in vitro* inhibitory activity on neutrophil pro-inflammatory responses, and *H. coronarium* has been found to be an active species. The MeOH extract of the rhizomes of *H. coronarium* showed potent inhibitory effects on superoxide anion generation and elastase release by human neutrophils in response to formyl-l-methionyl-l-leucyl-l-phenylalanine/cytochalasin B (fMLP/CB). Figure 1 illustrates the structures of four new labdane-type diterpenoids: hedychicoronarin (**1**), peroxycoronarin D (**2**), 7β-hydroxycalcaratarin A (**3**), and (*E*)-7β-hydroxy-6-oxo-labda-8(17),12-diene-15,16-dial (**4**). Thirteen known compounds (**5**–**17**), have been isolated and identified from the rhizomes of *H. coronarium* and their structures are depicted in Figure 2.

This paper describes the structural elucidation of the compounds numbered **1** through **4**, and the inhibitory activities of all isolates on superoxide generation and elastase release by neutrophils.

## 2. Results and Discussion

**Figure 3:**
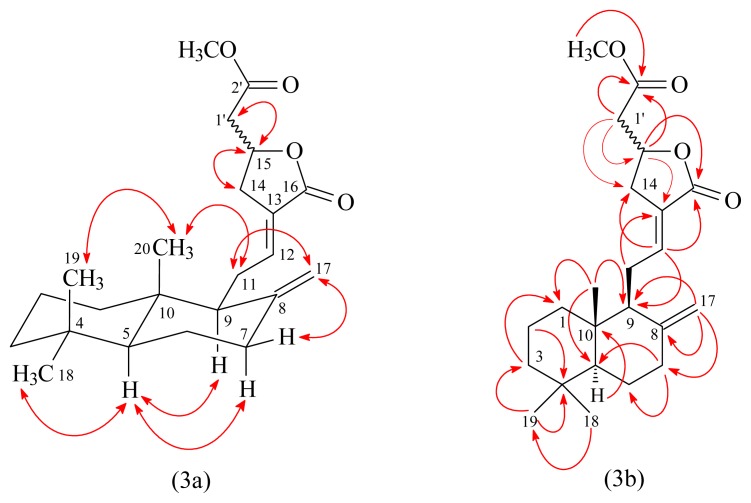
Key NOESY (3a) and HMBC (3b) correlations of **1**.

Hedychicoronarin (**1**) was isolated as an optically active colorless oil ([α]_D_^25^ = +13.8) with molecular formula C_23_H_34_O_4_ as determined by positive-ion HRESIMS, showing an [M + Na]^+^ ion at *m*/*z* 397.2353 (calcd for C_23_H_34_O_4_Na: 397.2355). The presence of two carbonyl groups was revealed by the bands at 1746 and 1757 cm^−1^ in the IR spectrum, and was confirmed by the resonances at δ 170.0 and 170.3 in the ^13^C-NMR spectrum. The ^1^H- and ^13^C-NMR spectra of **1** showed signals assignable to a methoxy [δ 3.72 (3H, s)], three methyls [δ 0.71, 0.81, 0.88 (each 3H, each s, H-20, 19, and 18)], an exo-methylene [δ 4.35 (1H, br s, H-17), 4.81 (1H, d, *J* = 1.2 Hz, H-17)], and an olefin [δ 6.72 (1H, m, H-12)], together with eight methylenes (H_2_-1, 2, 3, 6, 7, 11, 14, and 1′), two methines (H-5 and 9), an oxymethine (H-15), and six quaternary carbons (C-4, 8, 10, 13, 16, and 2′). The ^1^H- and ^13^C-NMR data of **1** was similar to those of coronarin D methyl ether (**8**) [10], except that the 2-methoxy-2-oxoethyl group [δ_H_ 2.64 (1H, dd, *J* = 16.0, 7.2 Hz, H-1’a), 2.85 (1H, dd, *J* = 16.0, 6.4 Hz, H-1’b), and 3.72 (3H, s, OMe-2′); δ_C_ 40.6 (C-1′), 52.0 (OMe), and 170.0 (C-2′)] at C-15 of **1** replaced the 15-methoxy group [δ_H_ 3.52/3.53 (3H, s, OMe-15); δ_C_ 56.54 (OMe-15)] of **8**. This was supported by (i) HMBC correlation observed between H-1′ (δ 2.64, 2.85) and C-14 (δ 31.3), C-15 (δ 73.0), and C-2′ (δ 170.0); and (ii) HMBC correlation observed between OMe-2′ (δ 3.72) and C-2′ (δ 170.0). The relative stereochemistry of **1** was elucidated on the basis of NOESY experiments (Figure 3). The NOESY cross-peaks between H-5/H-7α, H-5/H-9, H-5/H_3_-18, H_2_-11/H_3_-20, and H_3_-19/H_3_-20 suggested that H-5, H-7α, H-9, and H_3_-18 are α-oriented, and H_3_-19 and H_3_-20 are β-oriented. The occurrence of epimers of labdane diterpenes at C-15 position has been previously reported [3,10–12]. These labdane diterpenes with C-15 substituent were usually isolated as C-15 epimeric mixtures, which could not be separated. The presence of duplicated resonances of ^13^C-NMR signals of **1** at C-7 (δ 37.74/37.76), C-8 (δ 148.03/148.06), C-9 (δ 56.12/56.15), C-10 (δ 39.40/39.41), C-12 (δ 143.16/143.18), and C-17 (δ 107.36/107.43) as in the cases of coronarin D [10] and coronarin D methyl ether [10] suggested that it was isolated as a C-15 epimeric mixture. On the basis of the evidence above, the structure of **1** was elucidated as methyl 2-((*E*)-5-oxo-4-(2-((1*S*,8a*S*)-5,5,8atrimethyl- 2-methylenedecahydronaphthalen-1-yl)ethylidene)tetrahydrofu-ran-2-yl)acetate, named hedychicoronarin. This was further confirmed by ^1^H–^1^H COSY and NOESY experiments (Figure 3). The assignment of ^13^C-NMR resonances was confirmed by DEPT, HSQC and HMBC techniques (Figure 3).

**Figure 4:**
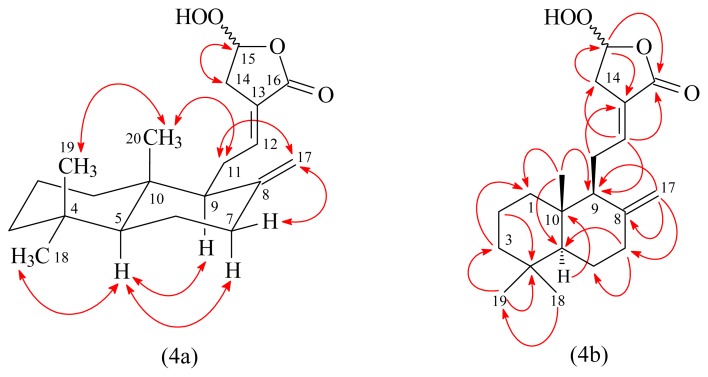
Key NOESY (4a) and HMBC (4b) correlations of **2**.

Peroxycoronarin D (**2**) was obtained as an optically active colorless oil ([α]_D_^25^ = +15.8). Its molecular formula, C_20_H_30_O_4_, was determined on the basis of the negative HRESIMS at *m*/*z* 333.2057 [M–H]^−^ (calcd 333.2066) and supported by the ^1^H, ^13^C, and DEPT NMR data. The IR spectrum showed the presence of OH (3413 cm^−1^), *exo*-methylene (3081, 1643, 889 cm^−1^), C=C (1678 cm^−1^), and carbonyl (1762 cm^−1^) groups. The ^1^H-NMR spectrum of **2** showed three methyl signals at δ_H_ 0.71, 0.81, and 0.88 (each 3H, each s, H-20, H-19, and H-18) and an exomethylene group at δ_H_ 4.41 and 4.82 (each 1H, each d, *J* = 1.2 Hz). These were characteristic of C-8 exomethylene labdane diterpenoids [13]. The ^1^H- and ^13^C-NMR data of **2** were similar to coronarin D (**7**) [10], except that the 15-hydroperoxy group of **2** replaced 15-hydroxy group of coronarin D (**7**). This was supported by (i) the MS (molecular weight of **2** was *m*/*z* +16 (O) more than coronarin D); (ii) the chemical shifts of C-15 (δ_C_ 100.4) of **2** appeared at relatively low field [C-15 (δ_C_ 95.9) of coronarin D], due to the electron*-*withdrawing effect of 15-OOH group of **2**; and (iii) the HMBC correlation (Figure 4) between H-15 (δ_H_ 5.61) and C-13 (δ_C_ 124.4), C-14 (δ_C_ 33.2), and C-16 (δ_C_ 169.9). However, the ^1^H- and ^13^C-NMR data of **2** showed two sets of signals at H-15 (δ_H_ 5.59/5.61), H_2_-17 (δ_H_ 4.34/4.41 and 4.80/4.82) and C-1 (δ_C_ 39.21/39.26), C-4 (δ_C_ 33.29/33.31), C-6 (δ_C_ 24.05/24.07), C-8 (δ_C_ 147.81/148.09), C-12 (δ_C_ 142.56/142.73), C-14 (δ_C_ 33.12/33.17), C-17 (δ_C_ 107.31/107.64), and C-20 (δ_C_ 14.30/14.34), which suggest that it was isolated as a C-15 epimeric mixture [3,10–12]. In addition, the relative stereostructure of **2** was elucidated by NOESY experiment, in which the NOESY correlations (Figure 4) were observed between the following proton pairs (H-5/H-7α, H-5/H-9, H-5/H_3_-18, H_2_-11/H_3_-20, and H_3_-19/H_3_-20). The full assignment of ^1^H and ^13^C-NMR resonances was confirmed by ^1^H–^1^H COSY, NOESY (Figure 4), DEPT, HSQC, and HMBC (Figure 4) techniques. Based on the data above, the structure of **2** was elucidated as (*E*)-5-hydroperoxy-3-(2-((1*S*,4a*S*,8a*S*)-5,5,8a-trimethyl-2-methylenedecahydronaphthalen-1-yl)ethylidene)dihydrofuran-2(3*H*)-one, named peroxycoronarin D.

**Figure 5:**
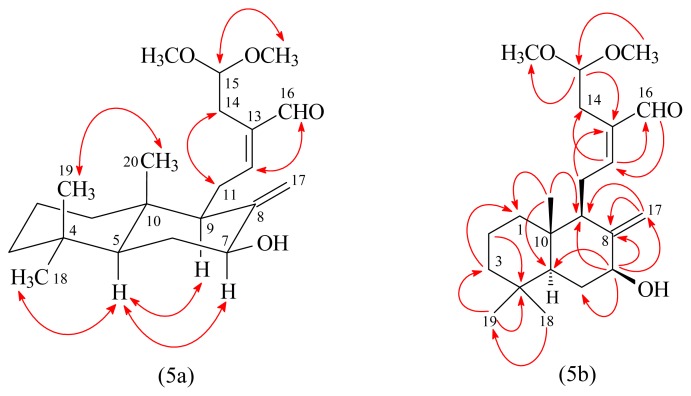
Key NOESY (5a) and HMBC (5b) correlations of **3**.

**Figure 6:**
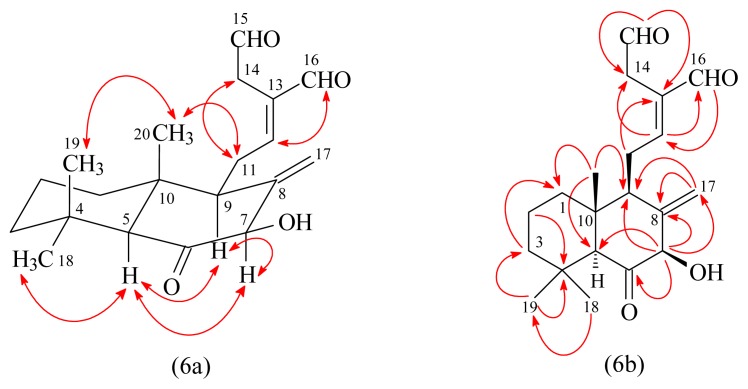
Key NOESY (6a) and HMBC (6b) correlations of **4**.

7β-Hydroxycalcaratarin A (**3**) was isolated as an optically active colorless oil ([α]_D_^25^ +11.9). The molecular formula C_22_H_36_O_4_ was deduced from a sodium adduct ion at *m*/*z* 387.2513 [M + Na]^+^ (calcd 387.2511) in the HRESI mass spectrum. The presence of hydroxy and carbonyl groups was revealed by the bands at 3429 and 1683 cm^−1^, respectively, in the IR spectrum. The ^1^H-NMR data of **3** was similar to those of calcaratarin A (**5**) [14], except that the 7β-hydroxy group of **3** replaced H-7β [δ 2.39 (1H, ddd, *J* = 12.9, 4.2, 2.4 Hz)] of calcaratarin A (**5**). This was supported by (i) the chemical shifts of H-7α (δ_H_ 4.00) and C-7 (δ_C_ 73.6) appeared at relatively low field, due to the electron*-*withdrawing effect of 7β-OH group; and (ii) the HMBC correlation (Figure 5) between H-7α (δ_H_ 4.00) and C-5 (δ_C_ 53.0), C-8 (δ_C_ 150.1), C-9 (δ_C_ 54.4), and C-17 (δ_C_ 104.4). The NOESY correlations between H-11 and H-14 and between H-12 and H-16 suggested 12*E*-configuration of **3**. The relative stereochemistry of **3** at chiral centers was based on the analysis of NOESY spectrum, which displayed NOESY correlations (Figure 5) between H-5/H-7, H-5/H-9, H-5/H_3_-18, and H_3_-19/H_3_-20 and suggested that H-5, H-7, H-9, and H_3_-18 are α-oriented, and H_3_-19, H_3_-20, and OH-7 are β-oriented. The structure elucidation of **3** was supported by ^1^H–^1^H COSY and NOESY (Figure 5) experiments, and ^13^C NMR assignments were confirmed by DEPT, HSQC, and HMBC (Figure 5) techniques.

(*E*)-7β-Hydroxy-6-oxo-labda-8(17),12-diene-15,16-dial (**4**) had the molecular formula C_20_H_28_O_4_, as indicated by the sodiated HRESIMS ion peak at *m*/*z* = 355.1882 [M + Na]^+^ (calcd for C_20_H_28_O_4_Na, 355.1885). IR absorption for a hydroxy function (3485 cm^−1^) was observed. The presence of three carbonyl groups was revealed by the bands at 1684, 1716, and 1728 cm^−1^ in the IR spectrum, which was confirmed by the resonances at δ 193.4, 197.2, and 207.9 in the ^13^C-NMR spectrum. The ^1^H-NMR spectrum of **4** showed three methyl signals at δ 0.68 (3H, s, H_3_-20), 1.00 (3H, s, H_3_-19), and 1.26 (3H, s, H_3_-18), and an exomethylene moiety at δ 4.67, 5.44 (each 1H, each br s, H_2_-17), which were characteristic of a labdane-type diterpenoid. Comparison of the ^1^H and ^13^C-NMR data of **4** with those of (*E*)-7β-hydroxy-labda-8(17),12-diene-15,16-dial [3] suggested that their structures were closely related except that the C-6 carbonyl group [δ_C_ 207.9] of **4** replaced the C-6 methylene group [δ_H_ 2.12 (1H, m, H-6α), 1.29 (1H, br t, *J* = 12.0 Hz, H-6β) and δ_C_ 33.7 (C-6)] of (*E*)-7β-hydroxy-labda-8(17),12-diene-15,16-dial. This was supported by HMBC correlation (Figure 6) observed between H-5 (δ 2.26) and C-6 (δ 207.9) and between H-7 (δ 4.50) and C-6 (δ 207.9). The NOESY correlation of the signal of the formyl group at δ_H_ 9.40 (H-16) and the olefinic signal at δ_H_ 6.75 (H-12) indicated this double bond to be in the *E*-configuration. The relative stereochemistry of **4** was elucidated on the basis of NOESY experiments (Figure 6). The NOESY cross-peaks between H-5/H-7, H-5/H-9, H-7/H-9, H-5/H_3_-18, and H_3_-19/H_3_-20 suggested that H-5, H-7, H-9, and H_3_-18 are α-oriented, and H_3_-19, H_3_-20, and OH-7 are β-oriented. The structure elucidation of **4** was further confirmed by ^1^H–^1^H COSY, NOESY (Figure 6), DEPT, HSQC, and HMBC experiments (Figure 6).

The known isolates were readily identified by a comparison of physical and spectroscopic data (UV, IR, ^1^H NMR, [α]_D_, and MS) with corresponding authentic samples or literature values, and this included six labdane-type diterpenoids, calcaratarin A (**5**) [14], coronarin A (**6**) [4], coronarin D (**7**) [4,10], coronarin D methyl ether (**8**) [15], coronarin D ethyl ether (**9**) [16], and (*E*)-labda-8(17),12-diene-15,16-dial (**10**) [4,5], five steroids, ergosta-4,6,8(14),22-tetraen-3-one (**11**) [17], a mixture of β-sitostenone (**12**) and β-stigmasta-4,22-dien-3-one (**13**) [18], and a mixture of 6β-hydroxystigmast-4-en-3-one (**14**) and 6β-hydroxystigmasta-4,22-dien-3-one (**15**) [19], and a mixture of stearic acid (**16**) and palmitic acid (**17**) [20].

Human neutrophils are known to play crucial roles in host defence against microorganisms and in pathogenesis of different diseases, such as rheumatoid arthritis, chronic obstructive pulmonary disease (COPD), asthma, and ischemia-reperfusion injury [21–24]. In response to diverse stimuli, activated neutrophils secrete a series of cytotoxins, such as the superoxide anion radical (O_2_^•−^), a precursor to other reactive oxygen species (ROS), granule proteases, bioactive lipids, and neutrophil elastase, a major contributor to destruction of tissue in chronic inflammatory disease [21,25,26]. Suppression of the extensive or inappropriate activation of neutrophils by drugs has been proposed as a method to ameliorate inflammatory diseases. The effects on neutrophil pro-inflammatory responses of compounds isolated from the rhizomes of *H. coronarium* were evaluated by suppressing fMet-Leu-Phe/cytochalasin B (fMLP/CB)-induced superoxide anion (O_2_^•−^) generation and elastase release by human neutrophils. The inhibitory activity data on neutrophil pro-inflammatory responses are summarized in Table 1. LY294002, a phosphatidylinositol-3-kinase inhibitor, was used as a positive control for superoxide anion generation and elastase release. Diphenyleneiodonium, an NADPH oxidase inhibitor, was used as a positive control for superoxide anion generation. From the results of our biological tests, the following conclusions can be drawn: (a) 7β-Hydroxycalcaratarin A (**3**), calcaratarin A (**5**), coronarin A (**6**), and (*E*)-labda-8(17),12-diene-15,16-dial (**10**) displayed potent inhibition (IC_50_ ≤ 4.52 μg/mL) of superoxide anion (O_2_^•−^) generation by human neutrophils in response to fMLP/CB. (b) 7β-Hydroxycalcaratarin A (**3**), (*E*)-7β-hydroxy-6-oxo-labda-8(17),12-diene-15,16-dial (**4**), calcaratarin A (**5**), coronarin A (**6**), (*E*)-labda-8(17),12-diene-15,16-dial (**10**), and ergosta-4,6,8(14),22-tetraen-3-one (**11**) exhibited potent inhibition (IC_50_ ≤ 6.17 μg/mL) against fMLP-induced elastase release. (c) Calcaratarin A (**5**), with 14-dimethoxymethyl group, exhibited more effective inhibition than its analogue, (*E*)-labda-8(17),12-diene-15,16-dial (**10**) (with 14-formyl group) against fMLP-induced O_2_^•−^ generation and elastase release. (d) Calcaratarin A (**5**), without any substituent at C-7, exhibited more effective inhibition than its analogue, 7β-hydroxycalcaratarin A (**3**) (with 7β-hydroxy group) against fMLP-induced O_2_^•−^ generation and elastase release. (e) (*E*)-Labda-8(17),12-diene-15,16-dial (**10**), without any substituent at C-6 and C-7, showed more effective inhibition than its analogue, (*E*)-7β-hydroxy-6-oxo-labda-8(17),12-diene-15,16-dial (**4**) (with 7β-hydroxy-6-oxo group) against fMLP-induced O_2_^•−^ generation and elastase release. (f) Among the labdane-type diterpenoid analogues (**1**, **2**, and **6–9**), coronarin A (**6**) (with a hydroxy group at C-7 and a furan-3-yl group at C-12) exhibited more effective inhibition than its analogues, **1**, **2**, and **7**–**9** (without any substituent at C-7 and with a 2-oxo-5-substituted-tetrahydrofuran-3-yl group at C-12) against fMLP-induced O_2_^•−^ generation and elastase release. (g) Calcaratarin A (**5**) displayed the most effective among the isolates, with IC_50_ values of 2.25 ± 0.42 and 2.36 ± 0.41 μg/mL, respectively, against fMLP-induced O_2_^•−^ generation and elastase release. Our study suggests *H. coronarium* and its isolates (especially **3**, **5**, **6**, and **10**) could be further developed as potential candidates for the treatment or prevention of various inflammatory diseases. Thus, the detailed mechanism of action of these compounds appears worthy of further investigation.

## 3. Experimental Section

### 3.1. General Experimental Procedures

Melting points were determined on a Yanaco micro-melting point apparatus and were uncorrected. Optical rotations were measured using a Jasco DIP-370 polarimeter in CHCl_3_. Ultraviolet (UV) spectra were obtained on a Jasco UV-240 spectrophotometer. Infrared (IR) spectra (neat or KBr) were recorded on a Perkin Elmer 2000 FT-IR spectrometer. Nuclear magnetic resonance (NMR) spectra, including correlation spectroscopy (COSY), nuclear Overhauser effect spectrometry (NOESY), heteronuclear multiple-bond correlation (HMBC), and heteronuclear single-quantum coherence (HSQC) experiments, were acquired using a Varian Unity 400 or a Varian Unity Plus-600 spectrometer operating at 400 or 600 MHz (^1^H) and 100 or 150 MHz (^13^C), respectively, with chemical shifts given in ppm (δ) using tetramethylsilane (TMS) as an internal standard. Electrospray ionisation (ESI) and high-resolution electrospray ionization (HRESI)-mass spectra were recorded on a Bruker APEX II or a VG Platform Electrospray ESI/MS mass spectrometer. Silica gel (70–230, 230–400 mesh, Merck) was used for column chromatography (CC). Silica gel 60 F-254 (Merck, Darmstadt, Germany) was used for thin-layer chromatography (TLC) and preparative thin-layer chromatography (PTLC).

### 3.2. Plant Material

The rhizomes of *H. coronarium* were collected from Taitung District Agricultural Research and Extension Station, Taitung County, Taiwan, in June 2009 and identified by J. F. Chen. A voucher specimen (Chen 3011) was deposited in the Department of Pharmacy, Tajen University, Pingtung, Taiwan.

### 3.3. Extraction and Isolation

The dried rhizomes (6.2 kg) of *H. coronarium* were extracted three times with MeOH (30 L each) for 3 days. The MeOH extracts were concentrated under reduced pressure at 35 °C, and the residue (638 g) was partitioned between *n*-hexane and H_2_O (1:1). The *n*-hexane layer was concentrated to give a residue (fraction A, 112 g). The water layer was further extracted with *n*-BuOH, and the *n*-BuOH-soluble part (fraction B, 245 g) and the water-solubles (fraction C, 258 g) were separated. Fraction A (112 g) was chromatographed on silica gel (70–230 mesh, 5.2 kg), eluting with *n*-hexane, gradually increasing the polarity with EtOAc and MeOH to give 11 fractions: A1 (2 L, *n*-hexane), A2 (2 L, *n*-hexane/EtOAc, 50:1), A3 (2 L, *n*-hexane/EtOAc, 30:1), A4 (3 L, *n*-hexane/EtOAc, 10:1), A5 (6 L, *n*-hexane/EtOAc, 5:1), A6 (3 L, *n*-hexane/EtOAc, 3:1), A7 (6 L, *n*-hexane/EtOAc, 1:1), A8 (5 L, *n*-hexane/EtOAc, 1:2), A9 (2 L, *n*-hexane/EtOAc, 1:4), A10 (5 L, EtOAc), A11 (2 L, MeOH). Fraction A4 (9.4 g) was separated by column chromatography on silica gel (230–400 mesh, 395 g) eluting with CHCl_3_/acetone (20:1) to yield 10 fractions (A4-1–A4-10). Fraction A4-4 (155 mg) was purified by preparative TLC (silica gel, *n*-hexane/EtOAc, 6:1) to afford a mixture of **12** and **13** (7.4 mg) (*R_f_* = 0.45). Fraction A4-5 (410 mg) was separated by MPLC (silica gel column, *n*-hexane/acetone, 8:1) to give 8 fractions (each 150 mL, A4-5-1–A4-5-8). Fraction A4-5-3 (98 mg) was purified by preparative TLC (silica gel, CHCl_3_/EtOAc, 50:1) to obtain **10** (3.8 mg) (*R_f_* = 0.56) and **11** (5.8 mg) (*R_f_* = 0.71). Fraction A5 (10.2 g) was chromatographed further on silica gel (230–400 mesh, 460 g) eluting with CHCl_3_/acetone (10:1) to give 12 fractions (each 1.2 L, A5-1–A5-12). Fraction A5-6 (510 mg) was purified by MPLC (silica gel column, 230–400 mesh, *n*-hexane/acetone, 5:1) to afford 10 fractions (each 160 mL, A5-6-1–A5-6-10). Fraction A5-6-5 (85 mg) was purified by preparative TLC (silica gel, CHCl_3_/EtOAc, 50:1) to afford **1** (4.2 mg) (*R_f_* = 0.58). Fraction A5-7 (87 mg) was purified by preparative TLC (silica gel, *n*-hexane/acetone, 10:1) to obtain **5** (2.5 mg) (*R_f_* = 0.57). Fraction A5-10 (162 mg) was purified by preparative TLC (silica gel, CHCl_3_/acetone, 50:1) to afford a mixture of **16** and **17** (5.7 mg) (*R_f_* = 0.50). Fraction A6 (9.5 g) was chromatographed further on silica gel (230–400 mesh, 430 g) eluting with *n*-hexane/acetone (8:1) to give 14 fractions (each 900 mL, A6-1–A6-14). Fraction A6-5 (132 mg) was purified by preparative TLC (silica gel, *n*-hexane/acetone, 6:1) to afford **3** (2.8 mg) (*R_f_* = 0.53). Fraction A6-6 (245 mg) was purified by preparative TLC (silica gel, *n*-hexane/EtOAc, 5:1) to obtain **6** (43 mg) (*R_f_* = 0.62) and **8** (57 mg) (*R_f_* = 0.45). Fraction A6-10 (175 mg) was purified by preparative TLC (silica gel, CH_2_Cl_2_/MeOH, 15:1) to yield a mixture of **14** and **15** (5.3 mg) (*R_f_* = 0.67). Fraction A7 (10.8 g) was chromatographed further on silica gel (230–400 mesh, 485 g) eluting with *n*-hexane/acetone (5:1) to give 12 fractions (each 850 mL, A7-1–A7-12). Fraction A7-4 (178 mg) was purified by preparative TLC (silica gel, *n*-hexane/acetone, 5:1) to afford **2** (2.6 mg) (*R_f_* = 0.71) and **7** (3.4 mg) (*R_f_* = 0.64). Fraction A7-5 (165 mg) was purified by preparative TLC (silica gel, CHCl_3_/MeOH, 15:1) to yield **9** (4.6 mg) (*R_f_* = 0.62). Fraction A7-6 (138 mg) was purified by preparative TLC (silica gel, CH_2_Cl_2_/MeOH, 12:1) to obtain **4** (3.1 mg) (*R_f_* = 0.59).

#### 3.3.1. Hedychicoronarin (**1**)

Colorless oil. [α]_D_^25^: +13.8 (*c* 0.12, CHCl_3_). UV (MeOH): λ_max_ (log ɛ) = 225 (4.06) nm. IR (neat): υ_max_ = 1757 (C=O), 1746 (C=O), 1676 (C=C), 3077, 1644, 887 (*exo*-methylene bonds) cm^−1. 1^H-NMR (CDCl_3_, 400 MHz): δ = 0.71 (3H, s, H-20), 0.81 (3H, s, H-19), 0.88 (3H, s, H-18), 1.07 (1H, ddd, *J* = 12.4, 12.4, 4.0 Hz, H-1α), 1.12 (1H, dd, *J* = 12.8, 2.8 Hz, H-5), 1.19 (1H, ddd, *J* = 13.2, 13.2, 4.2 Hz, H-3α), 1.33 (1H, dddd, *J* = 13.2, 12.8, 12.8, 4.4 Hz, H-6β), 1.41 (1H, br d, *J* = 13.2 Hz, H-3β), 1.51 (1H, m, H-2α), 1.58 (1H, ddddd, *J* = 13.2, 13.2, 12.4, 3.6, 3.2 Hz, H-2β), 1.68 (1H, br d, *J* = 12.4 Hz, H-1β), 1.73 (1H, dddd, *J* = 12.8, 5.0, 2.8, 2.4 Hz, H-6α), 1.86 (1H, br d, *J* = 10.8 Hz, H-9), 1.99 (1H, ddd, *J* = 13.2, 12.8, 5.0 Hz, H-7α), 2.38 (1H, ddd, *J* = 12.8, 4.4, 2.4 Hz, H-7β), 2.56 (1H, br d, *J* = 16.8 Hz, H-14), 2.64 (1H, dd, *J* = 16.0, 7.2 Hz, H-1’a), 2.85 (1H, dd, *J* = 16.0, 6.4 Hz, H-1’b), 3.10 (1H, dd, *J* = 16.8, 8.2 Hz, H-14), 3.72 (3H, s, OCH_3_), 4.35 (1H, br s, H-17a), 4.81 (1H, d, *J* = 1.2 Hz, H-17b), 4.91 (1H, ddd, *J* = 8.2, 7.2, 6.4 Hz, H-15), 6.72 (1H, m, H-12). ^13^C-NMR (CDCl_3_, 100 MHz, doubled signals due to epimers at C-15): δ = 19.3 (C-2), 24.1 (C-6), 25.5 (C-11), 31.3 (C-14), 33.5 (C-4), 37.74/37.76 (C-7), 39.3 (C-1), 39.40/39.41 (C-10), 40.6 (C-1′), 42.0 (C-3), 55.3 (C-5), 56.12/56.15 (C-9), 73.0 (C-15), 124.6 (C-13), 143.16/143.18 (C-12), 148.03/148.06 (C-8), 170.0 (C-2′), 170.3 (C-16). ESI-MS: *m*/*z* = 397 [M + Na]^+^. HR-ESI-MS: *m*/*z* = 397.2353 [M + Na]^+^ (calcd for C_23_H_34_O_4_Na: 397.2355).

#### 3.3.2. Peroxycoronarin D (**2**)

Colorless oil. [α]_D_^25^: +15.8 (*c* 0.14, CHCl_3_). UV (MeOH): λ_max_ (log ɛ) = 224 (4.01) nm. IR (neat): υ_max_ 3413 (OH), 1762 (C=O), 1678 (C=C), 3081, 1643, 889 (*exo*-methylene bonds) cm^−1. 1^H-NMR (CDCl_3_, 400 MHz): δ = 0.71 (3H, s, H-20), 0.81 (3H, s, H-19), 0.88 (3H, s, H-18), 1.06 (1H, ddd, *J* = 13.0, 12.6, 4.0 Hz, H-1α), 1.12 (1H, m, H-5), 1.18 (1H, ddd, *J* = 13.6, 12.8, 4.0 Hz, H-3α), 1.32 (1H, dddd, *J* = 13.0, 13.0, 12.8, 4.2 Hz, H-6β), 1.40 (1H, br d, *J* = 13.6 Hz, H-3β), 1.49 (1H, m, H-2α), 1.57 (1H, ddddd, *J* = 14.0, 12.8, 12.6, 3.0, 3.0 Hz, H-2β), 1.68 (1H, br d, *J* = 13.0 Hz, H-1β), 1.73 (1H, m, H-6α), 1.85 (1H, br d, *J* = 10.4 Hz, H-9), 1.99 (1H, ddd, *J* = 13.0, 12.8, 5.0 Hz, H-7α), 2.17 (1H, m, H-11), 2.33 (1H, m, H-11), 2.38 (1H, ddd, *J* = 12.8, 4.2, 2.4 Hz, H-7β), 2.66 (1H, br d, *J* = 17.4 Hz, H-14), 2.98 (1H, br d, *J* = 17.4 Hz, H-14), 4.34/4.41 (1H, d, *J* = 1.2 Hz, H-17a of C-15 epimers), 4.80/4.82 (1H, d, *J* = 1.2 Hz, H-17b of C-15 epimers), 5.59/5.61 (1H, dd, *J* = 2.0, 1.2 Hz, H-15 of C-15 epimers), 6.72 (1H, m, H-12). ^13^C-NMR (CDCl_3_, 100 MHz, doubled signals due to epimers at C-15): δ = 14.30/14.34 (C-20), 19.3 (C-2), 21.7 (C-18), 24.05/24.07 (C-6), 25.4 (C-11), 33.12/33.17 (C-14), 33.29/33.31 (C-4), 33.5 (C-19), 37.8 (C-7), 39.21/39.26 (C-1), 39.4 (C-10), 42.0 (C-3), 55.28/55.34 (C-5), 56.1 (C-9), 100.29/100.40 (C-15), 107.31/107.64 (C-17), 124.4 (C-13), 142.56/142.73 (C-12), 147.81/148.09 (C-8), 169.87/169.90 (C-16). ESI-MS: *m*/*z* = 333 [M − H]^−^. HR-ESI-MS: *m*/*z* = 333.2057 [M − H]^−^ (calcd for C_20_H_29_O_4_: 333.2066).

#### 3.3.3. 7β-Hydroxycalcaratarin A (**3**)

Colorless oil. [α]_D_^25^: +11.9 (*c* 0.15, CHCl_3_). UV (MeOH): λ_max_ (log ɛ) = 234 (4.01) nm. IR (KBr): υ_max_ = 3429 (OH), 1683 (C=O) cm^−1. 1^H-NMR (CDCl_3_, 600 MHz): δ = 0.73 (3H, s, H-20), 0.83 (3H, s, H-19), 0.92 (3H, s, H-18), 1.08 (1H, ddd, *J* = 13.2, 12.6, 4.2 Hz, H-1α), 1.17 (1H, dd, *J* = 12.6, 2.4 Hz, H-5), 1.21 (1H, ddd, *J* = 13.8, 13.2, 4.2 Hz, H-3α), 1.30 (1H, ddd, *J* = 12.8, 12.6, 11.4 Hz, H-6β), 1.45 (1H, br d, *J* = 13.2 Hz, H-3β), 1.53 (1H, m, H-2α), 1.58 (1H, m, H-2β), 1.73 (1H, br d, *J* = 12.6 Hz, H-1β), 1.88 (1H, br d, *J* = 10.8 Hz, H-9), 2.11 (1H, ddd, *J* = 12.8, 5.4, 2.4 Hz, H-6α), 2.46 (1H, ddd, *J* = 17.4, 10.8, 6.0 Hz, H-11), 2.57 (1H, dd, *J* = 13.2, 5.4 Hz, H-14), 2.60 (1H, dd, *J* = 13.2, 5.4 Hz, H-14), 2.64 (1H, ddd, *J* = 17.4, 6.0, 3.0 Hz, H-11), 3.35 (6H, s, OMe-15 × 2), 4.00 (1H, dd, *J* = 11.4, 5.4 Hz, H-7), 4.45 (1H, t, *J* = 5.4 Hz, H-15), 4.57 (1H, br s, H-17), 5.20 (1H, br s, H-17), 6.56 (1H, t, *J* = 6.0 Hz, H-12), 9.34 (1H, s, H-16). ^13^C-NMR (CDCl_3_, 150 MHz): δ = 14.4 (C-20), 19.3 (C-2), 21.6 (C-19), 24.8 (C-11), 29.0 (C-14), 33.5 (C-4), 33.5 (C-6), 33.6 (C-18), 39.1 (C-1), 39.2 (C-10), 41.9 (C-3), 53.0 (C-5), 54.3 (OMe-15 × 2), 54.4 (C-9), 73.6 (C-7), 103.9 (C-15), 104.4 (C-17), 138.1 (C-13), 150.1 (C-8), 160.0 (C-12), 195.0 (C-16). ESI-MS: *m*/*z* = 387 [M + Na]^+^. HR-ESI-MS: *m*/*z* = 387.2513 [M + Na]^+^ (calcd for C_22_H_36_O_4_Na: 387.2511).

#### 3.3.4. (*E*)-7β-Hydroxy-6-oxo-labda-8(17),12-diene-15,16-dial (**4**)

Colorless oil. [α]_D_^25^: +22.6 (*c* 0.21, CHCl_3_). UV (MeOH): λ_max_ (log ɛ) = 234 (3.97) nm. IR (neat): υ_max_ = 3485 (OH), 1728 (C=O), 1716 (C=O), 1684 (C=O) cm^−1. 1^H-NMR (CDCl_3_, 400 MHz): δ = 0.68 (3H, s, H-20), 1.00 (3H, s, H-19), 1.16 (1H, ddd, *J* = 13.6, 13.2, 4.4 Hz, H-3α), 1.26 (3H, s, H-18), 1.27 (1H, ddd, *J* = 13.2, 12.4, 4.0 Hz, H-1α), 1.43 (1H, br d, *J* = 13.2 Hz, H-3β), 1.55–1.63 (2H, m, H-2α and H-2β), 1.79 (1H, br d, *J* = 12.4 Hz, H-1β), 2.26 (1H, br s, H-5), 2.38 (1H, br d, *J* = 10.8 Hz, H-9), 2.39 (1H, ddd, *J* = 16.8, 10.8, 6.4 Hz, H-11), 2.52 (1H, ddd, *J* = 16.8, 6.4, 3.0 Hz, H-11), 3.44 (2H, br s, H-14), 4.50 (1H, br s, H-7), 4.67 (1H, br s, H-17), 5.44 (1H, br s, H-17), 6.75 (1H, t, *J* = 6.4 Hz, H-12), 9.40 (1H, s, H-16), 9.65 (1H, br s, H-15). ^13^C-NMR (CDCl_3_, 100 MHz): δ = 15.6 (C-20), 18.9 (C-2), 21.9 (C-19), 24.4 (C-11), 32.5 (C-18), 32.8 (C-4), 38.8 (C-1), 39.3 (C-14), 42.2 (C-3), 42.4 (C-10), 53.7 (C-9), 64.1 (C-5), 79.9 (C-7), 107.8 (C-17), 135.1 (C-13), 145.7 (C-8), 159.0 (C-12), 193.4 (C-16), 197.2 (C-15), 207.9 (C-6). ESI-MS: *m*/*z* = 355 [M + Na]^+^. HR-ESI-MS: *m*/*z* = 355.1882 [M + Na]^+^ (calcd for C_20_H_28_O_4_Na: 355.1885).

### 3.4. Biological Assay

The effect of the isolated compounds on neutrophil pro-inflammatory response was evaluated by monitoring the inhibition of superoxide anion generation and elastase release in fMLP/CB-activated human neutrophils in a concentration-dependent manner. The purity of the tested compounds was >98% as identified by NMR and MS. LY294002 (purity >99%, Sigma, St. Louis, MO, USA) was used as a positive control.

#### 3.4.1. Preparation of Human Neutrophils

Human neutrophils from venous blood of healthy, adult volunteers (20–28 years old) were isolated using a standard method of dextran sedimentation prior to centrifugation in a Ficoll Hypaque gradient and hypotonic lysis of erythrocytes [27]. Purified neutrophils containing >98% viable cells, as determined by the trypan blue exclusion method [28], were re-suspended in a calcium (Ca^2+^)-free HBSS buffer at pH 7.4 and were maintained at 4 °C prior to use.

#### 3.4.2. Measurement of Superoxide Anion Generation

The assay for measurement of superoxide anion generation was based on the SOD-inhibitable reduction of ferricytochrome *c* [29,30]. In brief, after supplementation with 0.5 mg/mL ferricytochrome *c* and 1 mM Ca^2+^, neutrophils (6 × 10^5^/mL) were equilibrated at 37 °C for 2 min and incubated with different concentrations (10–0.01 μg/mL) of compounds or DMSO (as control) for 5 min. Cells were incubated with cytochalasin B (1 μg/mL) for 3 min prior to the activation with 100 nM formyl-l-methionyl-l-leucyl-l-phenylalanine for 10 min. Changes in absorbance with the reduction of ferricytochrome *c* at 550 nm were continuously monitored in a double-beam, six-cell positioner spectrophotometer with constant stirring (Hitachi U-3010, Tokyo, Japan). Calculations were based on differences in the reactions with and without SOD (100 U/mL) divided by the extinction coefficient for the reduction of ferricytochrome *c* (ɛ = 21.1/mM/10 mm).

#### 3.4.3. Measurement of Elastase Release

Degranulation of azurophilic granules was determined by measuring elastase release as described previously [30]. Experiments were performed using MeO-Suc-Ala-Ala-Pro-Val-*p*-nitroanilide as the elastase substrate. Briefly, after supplementation with MeO-Suc-Ala-Ala-Pro-Val-*p*-nitroanilide (100 μM), neutrophils (6 × 10^5^/mL) were equilibrated at 37 °C for 2 min and incubated with compounds for 5 min. Cells were stimulated with fMLP (100 nM)/CB (0.5 μg/mL), and changes in absorbance at 405 nm were monitored continuously in order to assay elastase release. The results were expressed as the percent of elastase release in the fMLP/CB-activated, drug-free control system.

#### 3.4.4. Statistical Analysis

Results are expressed as the mean ± SEM, and comparisons were made using Student’s *t*-test. A probability of 0.05 or less was considered significant. The software SigmaPlot was used for the statistical analysis.

## 4. Conclusions

Seventeen compounds, including four new labdane-type diterpenoids **1**–**4**, were isolated from the rhizome of *H. coronarium*. The structures of these compounds were established on the basis of spectroscopic data. Reactive oxygen species (ROS) [e.g., superoxide anion (O_2_^•−^), hydrogen peroxide] and granule proteases (e.g., elastase, cathepsin G) produced by human neutrophils contribute to the pathogenesis of inflammatory diseases. The effects on neutrophil pro-inflammatory responses of isolates were evaluated by suppressing fMLP/CB-induced O_2_^•−^ generation and elastase release by human neutrophils. The results of anti-inflammatory experiments indicate that compounds **3**–**6**, **10**, and **11** can significantly inhibit fMLP-induced O_2_^•−^ generation and/or elastase release. Among the isolates, calcaratarin A (**5**) was the most effective among the isolated compounds, with IC_50_ values of 2.25 ± 0.42 and 2.36 ± 0.41 μg/mL, respectively, against fMLP-induced O_2_^•−^ generation and elastase release. The above isolated compounds might support the traditional use of *H. coronarium* for the treatment of inflammatory processes. Our study suggests *H. coronarium* and its isolates (especially **3**, **5**, **6**, and **10**) could be further developed as potential candidates for the treatment or prevention of various inflammatory diseases.

## Figures and Tables

**Table 1:** Inhibitory effects of compounds **1**–**17** from the rhizome of *H. coronarium* on superoxide radical anion generation and elastase release by human neutrophils in response to fMet-Leu-Phe/cytochalasin B a.

Compounds	Superoxide anion	Elastase
	IC_50_ (μg/mL) b	IC_50_ (μg/mL) b
Hedychicoronarin (**1**)	>10	>10
Peroxycoronarin D (**2**)	>10	>10
7β-Hydroxycalcaratarin A (**3**)	4.52 ± 1.14 e	3.86 ± 0.98 g
(*E*)-7β-Hydroxy-6-oxo-labda-8(17),12-diene-15,16-dial (**4**)	>10	5.36 ± 1.23
Calcaratarin A (**5**)	2.25 ± 0.42 e	2.36 ± 0.41 e
Coronarin A (**6**)	3.31 ± 2.41	2.67 ± 1.68
Coronarin D (**7**)	>10	>10
Coronarin D methyl ether (**8**)	>10	>10
Coronarin D ethyl ether (**9**)	>10	>10
(*E*)-Labda-8(17),12-diene-15,16-dial (**10**)	3.21 ± 1.24	3.70 ± 1.18 f
Ergosta-4,6, 8(14),22-tetraen-3-one (**11**)	>10	6.17 ± 1.30 e
Mixture of β-sitostenone (**12**) and β-stigmasta-4,22-dien-3-one (**13**)	>10	>10
Mixture of 6β-hydroxystigmast-4-en-3-one (**14**) and 6β-hydroxystigmasta-4,22-dien-3-one (**15**)	>10	>10
Mixture of stearic acid (**16**) and palmitic acid (**17**)	>10	>10
LY294002 c	0.36 ± 0.04	0.63 ± 0.07
Diphenyleneiodonium d	0.55 ± 0.24	

a Results are presented as averages ± SEM (*n* = 4).

b Concentration necessary for 50% inhibition (IC_50_).

c LY294002, a phosphatidylinositol-3-kinase inhibitor, was used as a positive control for superoxide anion generation and elastase release.

d Diphenyleneiodonium, an NADPH oxidase inhibitor, was used as a positive control for superoxide anion generation.

e *p* < 0.05 compared with the control.

f *p* < 0.01 compared with the control.

g *p* < 0.001 compared with the control.
